# Predictors of atrial emptying function in patients with hypertrophic cardiomyopathy: insights from cardiovascular magnetic resonance

**DOI:** 10.1186/1532-429X-14-S1-P163

**Published:** 2012-02-01

**Authors:** Radu Tanacli, Sacha Bull, Ntobeko Ntusi, Vanessa Ferreira, Jane M Francis, Saul Myerson, Hugh Watkins, Barbara Casadei, Stefan Neubauer, Theodoros Karamitsos

**Affiliations:** 1 Department of Cardiovascular Medicine, University of Oxford, Oxford, UK

## Background

Atrial function is an integral part of cardiac function that is often neglected. Previous studies have shown that the left atrium (LA) is dilated and its contractile capacity is reduced in HCM patients. Whether this is related to pathophysiological aspects of HCM such as the degree of left ventricular (LV) hypertrophy, myocardial fibrosis or diastolic dysfunction remains to be established.

## Methods

56 patients with HCM (44M/12F, mean age 55±14 years) and 50 normal controls (35M/15F, mean age 52±11 years) underwent CMR (Siemens Avanto 1.5T). HCM patients with reduced ejection fraction (EF<55%), known coronary artery disease, atrial fibrillation, hypertension, and moderate or severe valve disease were excluded. The imaging protocol included standard long and short-axis SSFP cines and late gadolinium enhancement (LGE) imaging. In addition to standard LV measurements (volumes, ejection fraction, mass), atrial volumes (max, min volume indexed for BSA) were measured using biplane-length-area method in 2 orthogonal planes (HLA and VLA SSFP retro-gated cine sequences). Diastolic function parameters (peak flow rate, time-to-peak-flow, deceleration time) were assessed using manual tracing of endocardial border across all temporal phases of the short axis stack and corrected for end-diastolic volume and RR interval respectively. Ventricular fibrosis was calculated using a full-width at half-maximum algorithm and expressed as LV mass percentage.

## Results

Maximum LA volume was increased (63.3±16.6 vs 50.8 ±14.5 ml/m2; p<0.001) and LA ejection fraction (LAEF) was significantly reduced (54.1±11.2% vs 63.9±4.8%; p<0.001) in HCM patients compared to normal controls, respectively (Figure). HCM patients had increased ejection fraction (76±6 vs 71±5%, p<0.001) and mass index (88±24 vs 57±12 g/m2, p<0.001) compared to normals. The average amount of fibrosis in HCM patients was 11±7%. Univariate and multivariate analysis for LAEF in HCM patients are summarised in table 1. Age, LV mass index, LV wall thickness, and myocardial fibrosis showed significant bivariate correlations with LAEF. To analyse the relation between LAEF and ventricular parameters we performed linear regression analysis. This showed that LAEF is predicted independently by 4 variables in a backward selection model (R2=0.415): age, LV mass index, extent of myocardial fibrosis, and the LV time to peak flow.

**Figure 1:**
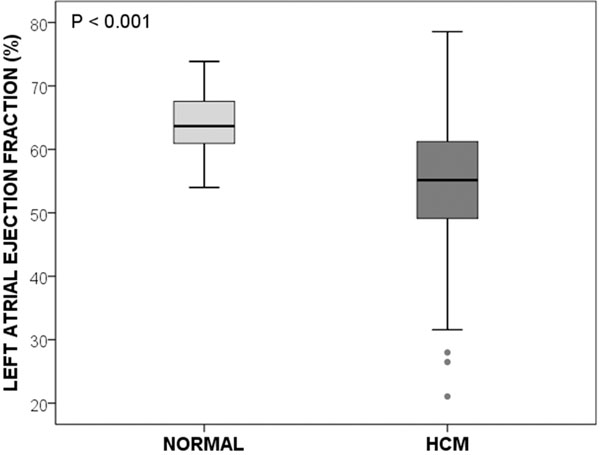
Left atrial ejection fraction in normal controls and HCM patients.

**Table 1:** Univariate and multivariate analysis of parameters associated with left atrial ejection fraction

VARIABLE	UNIVARIATE β	P value	MULTIVARIATE β	P value
Gender	0.081	0.281	n/a	n/a
Age	-0.292	0.017	-0.271	0.018
LV ejection fraction	0.140	0.158	n/a	n/a
LV wall thickness	-0.250	0.035	n/a	n/a
Wall thickness ratio	0.035	0.401	n/a	n/a
LV Mass Index	-0.489	<0.001	-0.470	<0.001
Peak flow rate	0.029	0.418	n/a	n/a
Time to Peak Flow	0.155	0.134	0.238	0.041
Deceleration Time	0.099	0.240	n/a	n/a
Fibrosis (% of LV mass)	-0.283	0.020	-0.243	0.038

## Conclusions

Atrial size is increased and function is impaired in HCM. This process is related to parameters characterizing other pathophysiological aspects of HCM including myocardial fibrosis, degree of LV hypertrophy, and LV diastolic dysfunction. In addition, age is an important contributor to LA emptying function.

## Funding

Dr Tanacli is funded by Dinu Patriciu Foundation Scholarship, Ratiu Family Foundation Grant. Prof Neubauer and Dr Karamitsos acknowledge support from the Oxford National Institute for Health Research Biomedical Research Centre Programme and the British Heart Foundation.

